# Three *Vibrio* isolates from the coral *Pachyseris speciosa* as draft genome resources

**DOI:** 10.1128/mra.00868-26

**Published:** 2026-10-14

**Authors:** Hannah Hau Ching Luk, Aaron An Rong Loh, Joao Paulo Andre Pereyra, Rebecca Josephine Case

**Affiliations:** 1 Singapore Centre for Environmental Life Sciences Engineering (SCELSE), Nanyang Technological University (NTU) 54761, Singapore, Singapore; 2 School of Biological Sciences, Nanyang Technological University (NTU) 54761, Singapore, Singapore; California State University San Marcos, San Marcos, California, USA

**Keywords:** coral, *Pachyseris speciosa*, *Vibrio harveyi*, *Vibrio coralliilyticus*, Singapore, equatorial coral reef, *Vibrio*

## Abstract

*Vibrio coralliilyticus* SCP4, *Vibrio harveyi* SCP5, and *Vibrio* sp. SCP2 were isolated from the coral *Pachyseris speciosa* in Singapore, adding to reports of *Vibrio* in tropical corals. Their draft genomes variably encode types II, III, and VI secretion systems, *toxR*, and *vcpA*, genes linked to pathogenic potential in *Vibrio* species.

## ANNOUNCEMENT

*Vibrio* species are well-recognized coral pathogens, prevalent in coral-associated bacterial communities in reef ecosystems (1). Isolation from equatorial coral reefs adds to the growing catalog of local environmental *Vibrio* isolates (2) and their potential for opportunistic pathogenicity (3).

*Pachyseris speciosa* samples were collected from the seawall at the northern side of Kusu Island, Singapore (1.2120°N, 103.8347°E) in August 2021, and maintained in an outdoor (0.8 m^3^) aquarium tank with seawater flow. Bacterial isolation methods were performed as described by Loh et al. (4). Briefly, coral tissue was separated from the skeleton by hydro-blasting, collected, and treated with proteinase K (Qiagen) in sterile artificial seawater, vortexed with mixed-sized (0.2–0.7 mm) glass beads in a bench top vortex (~200–2,700 rpm), and plated on marine broth agar (Difco 2216, BD). SCP2 and SCP4 were isolated at 33°C, and SCP5 at 28°C (Table 1). Colonies were subcultured under the same respective culturing conditions until pure isolates were obtained, then a single colony of each isolate was inoculated into marine broth and grown aerobically overnight.

**TABLE 1:** Genome features and GenBank accession numbers of the three *Vibrio* spp. isolates^*a*^

Isolate	*Vibrio* sp. SCP2	*Vibrio* sp. SCP4	*Vibrio* sp. SCP5
Source	Tissue	Tissue	Tissue
Growth temperature (°C)	33	33	28
Genome size (bp)	4,801,857	5,280,947	5,709,137
No. of CDS	4,375	4,761	5,079
No. of rRNAs	29	22	25
No. of tRNAs	99	102	123
G + C content (%)	44.9	45.4	44.9
No. of contigs	56	56	90
*N*_50_ (bp)	488,764	931,604	635,040
*L*_50_	3	2	3
Assembly accession no.	JCAFVV000000000	JCAFVU000000000	JCAFVT000000000
SRA accession no.	SRR39408556	SRR39408555	SRR39408554
No. of reads	9,433,035	11,343,331	14,057,343

^*a*^ CDS, coding DNA sequences.

Genomic DNA was extracted with the DNeasy PowerSoil Pro Kit (Qiagen, Hilden, Germany), using a Qubit fluorometer for quantification and quality control (ThermoFisher, USA). DNA library preparation and sequencing were performed by the SCELSE Sequencing Facility, NTU. DNA library preparation used the Accel-NGS 2S Plus Low Input DNA Library Prep Kit (Swift Biosciences, Ann Arbor, USA). Sequencing was performed using an Illumina HiSeq X Ten platform, version 2.5 (Illumina, San Diego, CA, USA), generating 150-bp paired-end reads.

Reads were quality-filtered and adaptor-trimmed using Trimmomatic version 0.39 (5). Default parameters were used unless specified. Draft genomes were assembled using the Shovill pipeline version 1.1.0 (https://github.com/tseemann/shovill) and the St. Petersburg genome Assembler version 3.15.5 (6). Annotations of the draft genomes were carried out with the Prokaryotic Genome Annotation Pipeline (PGAP) version 6.11 (7). No plasmids were detected by PlasmidFinder version 2.0.1 (8) in any of the assemblies, whose genome features are listed in Table 1.

Taxonomic classification using the Genome Taxonomy Database Toolkit v1.7.0 (9) identified *V. tubiashii* (GCF000772105), *V. coralliilyticus* (GCF000176135), and *V. harveyi* (GCF001591145) as the closest reference genomes to SCP2, SCP4, and SCP5, respectively, based on topology and average nucleotide identity (ANI). Phylogenetic relatedness between each draft genome and all publicly available reference genomes was assessed using the Orthologous Average Nucleotide Identity Tool, version 0.93.1 (10), and the Genome-To-Genome Distance Calculator, version 3.0 (11), to calculate the ANI and digital DNA-DNA hybridization (dDDH) scores, respectively. All draft genomes were <95% ANI and <70% DDH to each other, with SCP4 and SCP5 showing >95% ANI and >70% dDDH to their respective reference genomes. SCP2 showed >95% ANI but <70% dDDH to its reference genome, below the species threshold (12).

Dimethylsulfoniopropionate (DMSP) demethylation (*dmdA*) or cleavage (*ddd* lyase) genes, despite being important in marine systems (13), were absent from the PGAP annotation of all three draft genomes. Type II and type VI secretion systems are found in all three isolates (14, 15), with a type III secretion system, additionally identified in SCP5 (16). Despite lacking *luxI-luxR* quorum sensing, all isolates encode *toxR* homologue, a master transcriptional regulator for virulence (17), and *vcpA* homologue, a metalloprotease capable of coral tissue degradation (18), identified by NCBI online web BLASTp (version 2.17.0) with query sequences against our draft genomes.

## Data Availability

The SRA data were deposited to NCBI, and the complete genome sequences were deposited to GenBank under accession numbers listed in Table 1.
